# B and T Cell Immunity in Tissues and Across the Ages

**DOI:** 10.3390/vaccines9010024

**Published:** 2021-01-06

**Authors:** Jayaum S. Booth, Franklin R. Toapanta

**Affiliations:** 1 Center for Vaccine Development and Global Health, University of Maryland School of Medicine, Baltimore, MD 21075, USA; jbooth@som.umaryland.edu; 2 Department of Pediatrics, University of Maryland School of Medicine, Baltimore, MD 21201, USA; 3 Department of Medicine, University of Maryland School of Medicine, Baltimore, MD 21201, USA

**Keywords:** B cells, T cells, tissue-resident memory cells, intestine, respiratory track, vaccines, mucosal immunity

## Abstract

B and T cells are key components of the adaptive immune system and coordinate multiple facets of immunity including responses to infection, vaccines, allergens, and the environment. In humans, B- and T-cell immunity has been determined using primarily peripheral blood specimens. Conversely, human tissues have scarcely been studied but they host multiple adaptive immune cells capable of mounting immune responses to pathogens and participate in tissue homeostasis. Mucosal tissues, such as the intestines and respiratory track, are constantly bombarded by foreign antigens and contain tissue-resident memory T (T_RM_) cells that exhibit superior protective capacity to pathogens. Also, tissue-resident memory B (B_RM_) cells have been identified in mice but whether humans have a similar population remains to be confirmed. Moreover, the immune system evolves throughout the lifespan of humans and undergoes multiple changes in its immunobiology. Recent studies have shown that age-related changes in tissues are not necessarily reflected in peripheral blood specimens, highlighting the importance of tissue localization and subset delineation as essential determinants of functional B and T cells at different life stages. This review describes our current knowledge of the main B- and T-cell subsets in peripheral blood and tissues across age groups.

## 1. Introduction

The principal components of the adaptive immune system include B and T lymphocytes, which are crucial for the establishment and maintenance of immune responses. These two lymphocyte lineages express receptors that recognize and respond to diverse antigens from pathogens, commensals, tumors, and the environment. The mouse model has been used extensively to elucidate the in vivo functional role and mechanisms of B and T cells in immunity and immunopathology. These works have yielded tremendous insights into the workings of the immune system [1,2]. However, significant differences in the development, activation, and response to challenge in both innate and adaptive immunity between mice and humans exist. In addition, there are differences in the balance of leukocyte subsets, pattern recognition receptors (PRR) (e.g., Toll-like receptors (TLR)), and cytokines [2]. In consequence, the data from mouse studies have seldom been translated to human biology. Thus, human studies are necessary to determine the unique mechanisms involved in the development of B- and T-cell immunity.

The human immune system has evolved multiple mechanisms to ensure the survival of the host from recurrent infections and is continuously adapting to the relatively long life of humans. However, these mechanisms are not constant throughout life and, in fact, evolve over time. For instance, the immune system goes from tolerogenic, in the fetus, to a ready-to-respond status in the infant, albeit with limitations due to immaturity. In the next stage of life, the immune system matures and expands to fully functional during growth into adulthood. Finally, the immune system functionality decreases in older adults (termed immunosenescence). It is, thus, important to study the roles of B and T cells at different life stages.

For practical and ethical reasons, most human studies utilize blood samples to query the immune response following vaccination or infection. However, blood provides only a snapshot of the complex immune mechanisms elicited and provide limited information of the immunological events occurring in other tissues. Every organ in the body is populated with B and T cells that vary in quantity, characteristics, and function. In fact, the majority of B and T cells in the body are found in lymphoid organs (e.g., bone marrow, spleen, tonsils, lymph nodes) and mucosal sites (e.g., lungs, intestine). Examples of the differences in immune cells between blood and tissues include: (1) human peripheral blood contains only ~2–3% of the total body T cells [3,4]. (2) The distribution of CD4+ and CD8+ T naïve and memory cells varies in tissues, compared to blood. Specifically, there is a greater number of effector memory T (T_EM_) cells in the small intestine than in peripheral blood. These observations, among others, suggest unique roles for T cells in immunity at specific anatomic compartments which are distinct from the periphery and that most likely can be extended to other cells of the immune system. Thus, it is important to evaluate B- and T-cell immunity in tissues, especially in anatomical regions with high antigenic exposure (e.g., lungs and intestine) and correlate their function with their peripheral counterparts.

In this review, we will describe human B- and T-cell differentiation and function following vaccination and/or infection and how their tissue localization impacts functionality and maintenance. We will also discuss these aspects as they relate to the aging process. All these facets are necessary to consider when developing strategies to modulate B- and T-cell immunity via vaccines and/or immunotherapies.

## 2. Immune Cells in Peripheral Blood: Main B- and T-cell Populations

### 2.1. B Cells: A Complex Population Composed of Various Subsets with Diverse Roles in Immunity

B cells constitute a critical arm of the immune system and are responsible for the short- and long-term generation of humoral antibody responses. B cells also perform antibody-independent functions such as: (1) antigen-presentation, (2) modulation of T-cell differentiation and survival, and (3) production of regulatory and pro-inflammatory cytokines [5,6,7,8]. The human mature B compartment encompasses (1) naïve B cells, (2) germinal center (GC) B cells, (3) memory B (B_M_) cells, and (4) antibody-secreting cells (ASC) [9]. Importantly, except for GC cells, all human B-cell populations found in lymphoid tissues can also be demonstrated in the peripheral blood. Below we describe the most important B-cell subsets and their role in the development of adaptive responses.

#### 2.1.1. Plasmablasts (PBs) 

Most studies consider human PBs as blood short-lived ASCs generated during acute B-cell responses to infection or vaccination. These cells burst into circulation 5–9 days after infection/vaccination and transiently contribute to the serum antibody [10,11,12,13]. In a secondary response to an antigen such as influenza virus vaccine, antigen-specific IgG-secreting PBs with evidence of somatically mutated VH gene rearrangements are generated, suggesting that these cells come from B_M_ cells [9,14,15,16,17]. Secondary PB responses also include clones with limited VH rearrangements suggesting that these come from newly selected antigen-specific B cells (naïve). However, these new clones appear to be a minority in secondary responses [17,18]. Therefore, PBs induced in secondary responses constitute a mixed population including cells from the B_M_ compartment and newly selected B-cell clones. Similar results have been published for other pathogens such as Ebola and Dengue virus infections [13,14]. Importantly, PBs induced by infection/vaccination are migratory and attracted by cytokines such as CXCL12 and can migrate to several tissues, such as the bone marrow. Our group has shown that oral immunization induces antigen-specific PBs expressing integrin α4β7 and CD62L suggesting their ability to migrate to gut-associated lymphoid tissues (GALT) as well as to secondary lymphoid organs (e.g., lymph nodes) [10]. This data also suggest that the route of immunization is an important factor driving the migration pattern of PBs [9,19]. Influenza studies that allowed characterization of PBs have also led to the identification of a subset of antigen-reactive B cells, called activated B cells (ABCs), that are transcriptionally distinct from PBs and committed to the memory lineage [18,20]. Importantly, ABCs and PBs shared hemagglutinin (HA)-reactive clones following influenza vaccination. It is expected that B cells reactive to other influenza antigens (e.g., NA) will follow a similar pattern.

#### 2.1.2. Long-Lived Plasma Cells (LLPC)

Humoral immunological memory is mediated in part by serum antibodies secreted by long-lived plasma cells (LLPCs). These cells reside in the human bone marrow where mesenchymal stromal cells (MSC) provide a microenvironment and other factors that support their survival [21,22,23]. Factors promoting plasma cells (PCs) survival include cytokines such as BAFF, APRIL, TNF-α, and IL-6. Ligands for CD80/CD86, CD44 binding to hyaluronic acid, and VLA-4 binding to VCAM-1/fibronectin also promote survival. CXCL12 is a critical cytokine, since it promotes entry of cells to the bone marrow and supports PCs survival [24]. Human LLPCs freshly isolated from the bone marrow express CXCR4, CXCR6, CCR10, and CCR3 [25]. In secondary immune responses PCs migrate from the bone marrow to the circulation as suggested by the identification of a PBs subset that resembles mature bone marrow PCs [9] and, as discussed above, contain clones with mutated VH chains. Importantly, as of today, the niches for survival of LLPC outside of the bone marrow remain poorly understood. Some studies have shown that stromal cells in the spleen and lymph nodes might promote PC survival in vitro [26,27]. Of note, a subset of fibroblasts (fibroblastic reticular cells -FCR-) able to guide PCs in their migration was described in the lymph nodes of mice and humans [28]. Finally, as discussed in sections below, the gut contains all elements required for PCs survival; thus, it is likely a niche for LLPCs.

#### 2.1.3. Memory B (B_M_) Cells

B_M_ cells are long-lived quiescent cells that can respond quickly to an antigenic recall [20,29,30,31]. Unlike LLPC, B_M_ cells do not produce antibodies constitutively [32,33,34]. There is a broad consensus that human B_M_ cells can be defined by the expression of CD19 and CD27. Importantly, CD19+ CD27+ cells in circulation also include antibody-secreting PBs (discussed above), which can be differentiated by the higher levels of expression of CD27 and CD38 (CD27++ CD38++). CD27 is used to define B_M_ cells because circulating CD27+ cells, like GC cells, which initiate upregulation of CD27 [35,36], show significant rates of somatic hypermutation [36,37,38]. CD27+ B cells also have an enhanced capacity to differentiate into ASC and show a distinct transcriptional program [39]. B_M_ cells are mainly generated in the GCs in secondary lymphoid organs. After leaving the GCs, B_M_ cells either join the recirculating pool of lymphocytes, or home to antigen-draining sites. B_M_ cell niches outside of the blood have been described and B_M_ cells have been found in the bone marrow, the tonsil, and the spleen [40]. Importantly, CD21 seems to be an important marker to distinguish activated cells within some B_M_ subsets since constitutive expression of CD21 is maintained in resting memory cells and lost upon activation as illustrated by substantial increases of CD21low CD27+ memory cells in HIV infection [41] and in patients with SLE and RA [42,43]. Activated memory cells also upregulate CD95, CD80, and CD86. Of note, upregulation of CD71 appears to be a helpful marker of early activation in proliferative antigen-specific memory cells and new germinal center products that differentiate into antibody-secreting PB [18,20,44].

#### 2.1.4. Atypical B Cells

Double negative (DN) B cells (CD19+ CD27- IgD-) accumulate in certain pathologies such as SLE, chronic HIV viremia, and malaria infections [41,45,46]. In normal healthy individuals DN cells represent ~10% of all CD19+ cells in peripheral blood lymphocytes; meanwhile during active SLE these cells can account for >40% of all circulating B cells and may become the largest circulating population of isotype-switched IgD- cells [43,47]. DN cells are largely IgG+ or IgA+ and display a significant degree of somatic hypermutation; however, at lower levels than CD27+ memory cells.

### 2.2. T Cells in Peripheral Blood: Effector and Memory Cells

Our understanding of T memory (T_M_) cells has advanced considerably during recent years. It is now clear that the T_M_ compartment is composed of multiple cell populations that show considerable heterogeneity due to their diverse anatomical location as well as functional, phenotypic, and developmental characteristics.

T-cell differentiation occurs in three phases. First, clonal expansion phase, whereby activated pathogen-specific T cells expand and differentiate into effector T cells that mediate infection clearance. Second, the contraction phase, where most of the effector T cells die by apoptosis following infection. Third, memory phase, where primed T cells persist as long-term T_M_ cells reactivated in re-infections [48,49]. T_M_ cell diversity is based on their effector function, location, surface-receptor expression, and trafficking properties. In human peripheral blood, T_M_ cells represent a major circulating population with at least four distinct subsets namely central memory T cells (T_CM_, CD62L+ CD45RA-), effector memory T cells (T_EM_, CD62L- CD45RA-), terminal effector memory T cells (T_EMRA_; CD62L- CD45RA+), and stem-like memory T cells (T_SCM_, CD45RA+ CCR7+ CD95+ CD122+) [50,51]. Upon stimulation, both T_CM_ and T_EM_ produce IL-2 and effector cytokines; however, T_CM_ have a greater proliferation capacity and a lymph node-homing profile while T_EM_ produce more effector cytokines [51,52]. T_SCM_ cells have self-renewable capacities and are highly proliferative but with no effector function [50]. There is evidence that memory subsets can interconvert between them [53] and that external factors such as inflammation, cytokines, signaling via the T-cell antigen receptor can be strong determinants of T lymphocyte differentiation [54]. Epigenomic profiling of CD4+ T cells has shown that key genes that control memory development are progressively lost in the order of naïve-T_CM_-T_EM_ [55]. Interestingly, for human CD8+ T cells, the differentiation is in the order of naïve-T_SCM_-T_CM_-T_EM_ as revealed by methylation analysis and chromatin accessibility studies [56,57]. In tissues, T_M_-cell subsets have different frequencies, phenotypes, and functional characteristics suggesting that there may be distinct expression patterns of genes that control memory development and allow their establishment in tissues.

There is also a wide diversity among the effector T-cell population. For example, CD4+ T_M_ can differentiate into various effector subsets such as helper T-cell subsets T_H_1, T_H_2, T_H_17, T_H_9, follicular helper T cells (T_FH_), and induced regulatory T cells (T_regs_) [58]. Each of these effectors have specialized function(s) and adapted to deal with specific pathogen and counterbalance excessive activation in order to maintain immune homeostasis [59]. Similarly, CD8+ T_M_ lymphocytes can differentiate into various effectors such as T_C_1, T_C_17 that can produce one or multiple cytokines and chemokines and/or expressed perforin, granzymes, and degranulation molecules. All of these functions correlate with improved protection against infection [60]. For example, in a *S*. Typhi controlled human infection model (CHIM), the number and frequency of multifunctional (MF) *S*. Typhi-specific effector and memory CD8+ subsets, at baseline, correlate with protection from disease [61]. In addition, numerous studies by our group have shown that the typhoid oral vaccine, Ty21a, elicits both CD4+ and CD8+ T-cell responses including IFN-γ, cytotoxic T cells (CTL), proliferation, and MF antigen-specific cytokine-producing cells [62,63,64,65,66,67,68], which might play an important role in long-term immunity. We have also reported that Ty21a elicits *S*. Typhi-specific CD4+ and CD8+ T-cell responses in PBMC by various T memory (T_M_) cell subsets, including T central/memory (T_CM_), T effector/memory (T_EM_), and CD45RA+ T_EM_ (T_EMRA_) [69,70]. However, all these detailed CMI responses were evaluated in peripheral blood.

### 2.3. T Follicular Helper (T_FH_) Cells

T follicular helper (T_FH_) cells are a subset of CD4+ T helper cells that expressed the transcription factor, Bcl-6 and on its surface, the chemokine receptor CXCR5. This receptor, CXCR5, enable the migration of T_FH_ into B-cell follicles within secondary lymphoid organs (SLO) and then provide efficient help to B cells to produce antibodies [71]. T_FH_ cells co-localize with B cells in the germinal center of SLO where they interact with B cells by cell contact and soluble signals that support survival and differentiation of B cells. The germinal center is where antigen-specific B cells proliferate, undergo immunoglobulin affinity maturation, class switch recombination, and differentiation into LLPCs and B_M_ cells. While most of the T_FH_ cells are found in the SLO, they are also found in circulating blood and consist of subsets that are essential for extrafollicular antibody responses and provide short-lived antibody-producing PBs and B_M_ cells [72,73,74]. A recent study detailed the similarities and differences between blood and tonsils T_FH_ and provided data that circulating T_FH_ cells might be the memory cell counterparts of bona fide T_FH_ cells in secondary lymphoid organs [75]. However, the authors found differences in terms of functional capacities within the extrafollicular stages. Therefore, T_FH_ cells have different functional properties in blood and tissues and are essential to the generation of short- and long-lived humoral immunity, necessary for the protective response against a wide range of pathogens.

Understanding key factors that regulate the heterogeneity of B cells as well as T effector/memory cells will greatly inform the development of novel vaccine strategies. However, in humans, most studies have assessed cells from peripheral blood and neglected the study of immune cells in compartments that house large numbers of these cells. Indeed, one of the most abundant T_M_-cell populations in tissues, especially in the mucosa, are the newly described tissue-resident memory T cells (T_RM_). In the next section, we discuss specialized B and T cells that are responsive and reside in tissues.

## 3. Immune Cells in the Gut

### 3.1. Gut-Associated Lymphoid Tissues (GALT): Structure and Function

**Figure 1:**
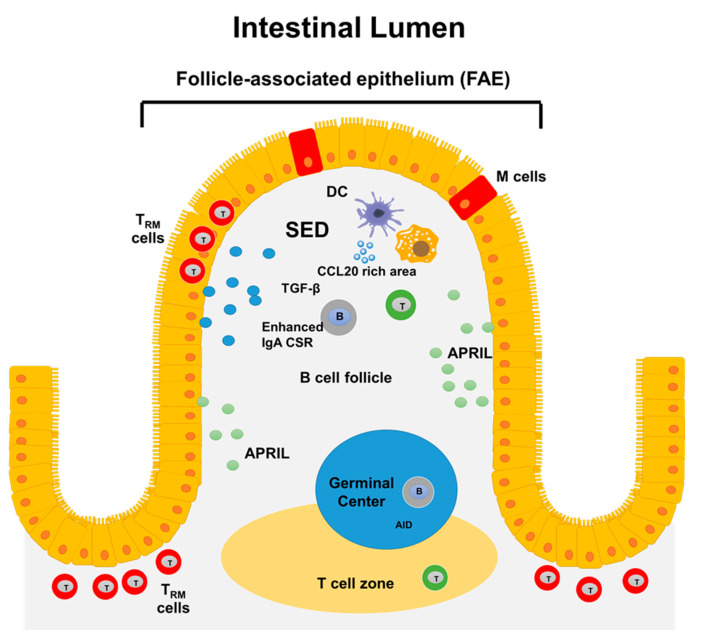
**Structure of GALT**. In the gut, antigens are sampled by follicle-associated epithelium (FAE) which contain microfold (M) cells. Next to M cells the sub-epithelial dome (SED) region is located. This region hosts multiple cell types, including different subsets of dendritic cells (DCs), macrophages, neutrophils, B, and T cells. Several factors that favor PCs survival (e.g., APRIL) and CSR to IgA (e.g., TGF-β) are present in GALT. The GALT also contains germinal centers associated with T-cell zones for induction of immune responses at the local level. Finally, the human gut mucosa contains tissue-resident memory T cells, which show evidence of previous antigen encounter and appear to be long-lived.

The gut contains specialized lymphoid tissues such as gut regional lymph nodes and micro-anatomically discrete zones of organized lymphoid known as GALT that include the Peyer’s Patches (PP). PP are particularly important since these are constantly exposed to a great diversity of antigens derived from food and microbes which causes PP to host many GCs [76]. Also, PP play important roles in the production of secretory IgA as well as in the development of immune responses to pathogens. The microanatomy of the PP includes B-cell follicular areas, inter-follicular regions enriched for T cells, and a follicle-associated epithelium (FAE), which acts as an interface between the lumen and the underlying sub-epithelial dome (SED) (Figure 1) [77]. The specialized microfold (M) epithelial cells of the FAE take up antigens derived from bacteria, viruses, fungi, toxins, inert particles, and immune complexes [78,79]. The uptake mechanism is facilitated by the expression of certain receptors, such as Dectin-1 and glycoprotein-2 (GP2) which bind whole bacteria or soluble antigens [80]. Next to M cells is the SED region that host multiple cell types, including different subsets of dendritic cells (DCs), macrophages, neutrophils, B and T cells [81]. At the basal side of the M cells, pocket containing DCs, T and B cells are formed [82,83]. Importantly, B and T cells localize at the SED only when they express CCR6 since this site produces CCL20 (CCR6 ligand) [77,84]. Studies have identified activated IgD+ B cells that are at a pre-GC stage in SED, where they express activation-induced cytidine deaminase (AID), and, therefore, could undergo class-switch recombination (CSR) to IgA1. Activated B cells start to express CCR6 and move into the SED where they interact with other cells, such as CD11b+ CD8− DCs [85,86]. These DCs express integrin αvβ8 that can activate latent-transforming growth factor β (TGFβ), the prime switch factor required for IgA CSR [87,88,89]. Interestingly, the key source of latent TGFβ appears to be B cells themselves. CD4 T cells, FDCs, and DCs can also produce this cytokine [81]. B cells proliferate in the SED, which currently is considered a prerequisite for CSR [39,90].

### 3.2. B Cells in the Gut

#### 3.2.1. Intestinal IgA functions and Lamina Propria PCs 

Immune protection of the human intestinal mucosa is highly dependent on secretory IgA (sIgA) and the body produces 3–5 g of this Ig. In the gut lumen, sIgA acts as a first-line barrier that limits the access of intestinal antigens to the blood circulation and controls the intestinal microbiota [91,92]. These functions of sIgA, collectively referred to as “immune exclusion,” involve various mechanisms including antigens entrapment in the mucus to prevent their binding to cell-surface receptors as is the case in sIgA-mediated neutralization of cholera toxin [93]. Moreover, sIgA can affect bacterial virulence, for instance sIgA binding reduces the motility of *Salmonella* spp. [94] and limits the invasiveness of *Shigella flexneri* [95]. sIgA can also aid in the uptake of luminal antigens across the intestinal epithelium into IgA-inducing lymphoid compartments [96]. IgA also play roles outside of the gut lumen, for example, IgA can neutralize antigens within epithelial cell endosomes and modulate immune functions by binding to Fcα receptors [97]. It is likely that IgA also aids in the excretion of antigens from the lamina propria into the gut lumen [98]. Importantly, IgA responses to pathogenic microorganisms can also be induced. Most IgA responses to pathogenic bacteria, toxins, and viruses are T-dependent and yield high-affinity antigen-specific IgA [76]. In summary, IgA reinforce the integrity of the intestinal barrier, dampen pro-inflammatory immune responses, effectively contribute to intestinal homeostasis and high-affinity antigen-specific IgA responses to pathogenic microorganisms can be also be induced.

The precursors of lamina propria IgA PCs are generated in the GALT and gut regional lymph nodes [99]. In duodenum/jejunum, 79% of PCs express IgA, 18% express IgM, and 3% express IgG. Meanwhile, in colon the corresponding percentages are 90, 6, and 4% [100]. Locally produced sIgA is mostly dimeric and bound together by the Joining (J)-chain which attaches to the Ig receptor (pIgR) for active transport into the gut lumen. Intestinal IgA include IgA1 and IgA2 subclasses and the ratio between PCs secreting these subclasses differs along the gastrointestinal tract. Whereas most PCs in the small intestine secrete IgA1, the proportion of IgA2 increases from the duodenum through to the terminal ileum. In the colon, IgA1 and IgA2 are present in approximately equal numbers. Of note, IgA2 has a shorter hinge than IgA1 and therefore it is less susceptible to bacterial proteases. In consequence, IgA2 has a functional advantage in the lumen of the colon. Intestinal PCs have low expression of CCR6 and CCR7 and high expression of CCR10 and CXCR4 [101]. The expression of integrin α4β7 in PCs is lower than that of blood ASCs. Finally, ASCs derived from B cells activated in GALT can circulate via the blood and home back to the gut. Homing is mediated by a combination of lymphocyte-homing receptors for endothelial ligands such as integrin α4β7 receptor for mucosal endothelial MAdCAM and chemokine receptors for chemokines secreted by intestinal epithelial cells such as CCR9 and CCR10 that facilitate migration toward CCL25 and CCL28, respectively [102].

#### 3.2.2. Tissue-Based Memory B Cells 

One distinctive feature of human GALT marginal zone B cells is the expression of Fc receptor-like 4 (FcRL4) [103]. This inhibitory receptor suggests that B cells on the mucosal front line in humans have distinctive raised thresholds of responsiveness [104,105]. FcRL4 also may detach B-cell responses from the activation signals normally driven through BCR ligation in favor of responses through innate receptors [106]. In healthy individuals, FcRL4 expressing B cells are rare in blood and lymphoid tissues distant from epithelia.

#### 3.2.3. Evidence for PC Survival Niches in the Intestine 

Bone marrow factors that allow LLPCs to home and survive for extended time periods were discussed above. There is evidence that the certain areas of intestine can provide an environment conductive of long-term survival of PCs. For example, culture of gut biopsies without tissue disruption results in high antibody production and PC survival suggesting that the tissue environment plays a role in gut PC longevity [107]. In cultures of these gut biopsies, IL-6 and APRIL were detected. Importantly, blocking the activity of endogenous APRIL and IL-6 reduced antibody secretion, which suggested a role for these cytokines in gut PC survival. Immunohistochemistry and quantitative rt-PCR showed that lamina propria macrophages, DCs and neutrophils expressed APRIL [108]. APRIL was also intensely expressed by crypt epithelial cells. Similarly, mRNA for the APRIL receptors TACI and BCMA were detected in micro-dissected lamina propria tissue. Flow cytometric analysis of celiac disease biopsies showed that BCMA was expressed on most PCs, whereas BAFF-R and TACI were expressed only by a subgroup of cells and at variable levels. IL-6 is produced by human small intestinal epithelial and smooth muscle cell lines in vitro and such cells could be sources of IL-6 in the intact intestinal tissue [109]. Immunohistochemical analysis suggests that CXCL12 is constitutively expressed in the intestine by enterocytes but not by stromal cells, and it is upregulated upon inflammation [110]. In summary, several elements required for PC long-term survival in the bone marrow are also present in the gut.

### 3.3. Intestinal Resident T Cells

The majority of T cells in the human body reside in tissues and do not recirculate; thus, these represent a distinct pool from those in circulation. These non-circulating T_M_ cells are termed resident memory T cells (T_RM_) and are abundant at the intestinal and lung mucosa. During their retention in tissues, T_M_ adapt and monitor distinct locations. These cells not only detect infections but also induce changes in immune homeostasis. Thus, it is important to understand the mechanisms involved in eliciting and maintaining long-term immunity of T_RM_ after vaccination/infection. As of today, it is not clear what fraction of the T_RM_ (CD4+ and CD8+ T_RM_) are in transition within the tissues and whether these cells can recirculate. In addition, while expression of homing molecules by T_M_ cells plays a critical role for tissue residence, it is still unclear how resident cells traffic, populate, and are maintained in different tissues (e.g., lungs vs. intestine).

#### 3.3.1. CD4+ T_RM_

CD4+ T cells are crucial for generating vaccine-mediated immune responses and both, circulating T_M_ and T_RM_ are central for eliciting protective immunity [111,112]. A key marker to distinguish between them is CD69, which is highly expressed by human T_RM_ [113]. The ligand to E-cadherin, integrin αEβ7 (CD103), is also used to characterize T_RM_ but its expression is mostly confined to CD8+T_RM_ and a minor subset of CD4+T_RM_ [113,114,115,116]. Recent studies have characterized CD4+ T_RM_ subsets in multiple organs including lungs, liver, skin, intestines, vagina, and lymphoid sites where they provide protective responses and direct the recruitment of immune cells [117,118,119,120]. However, the exact molecular mechanism for eliciting CD4+ T_RM_ in specific tissues remains unclear. There are indications that cytokines such as IL-2, IL-15, and IL-7, which play fundamental roles in circulating CD4+ T cell’s biology [121], are also involved in CD4+ T_RM_’s biology in tissues, albeit differently. For example, IL-2 receptor mediated signals are essential for the generation of CD4+ T_RM_; however, sustained IL-2 in circulating CD4+ inhibit their fate toward the memory phenotype [122,123]. IL-2 independent CD4+ T_RM_ cells have also been identified. These cells require IL-15, which acts as an “alarm” at local sites of infection, to promote both CD8^+^ T-cell responses and induce long-lived CD4^+^ T_RM_ [124,125]. Maintenance of both naïve and lymphoid homing CD4+ T_M_ cells requires IL-7 signaling [126]. Altogether, these data suggest an important role for IL-2, IL-15, and IL-7 signaling in the recruitment, formation, and maintenance of CD4+ T_RM_ in many tissues. In addition to cytokines, TCR antigen recognition is required for CD4+ T cells to differentiate into effector and memory subsets. However, in CD4+ T_RM_ maintenance, the role of antigen recognition is less clear. Interestingly, CD4+ T_RM_ form clusters in close proximity with other resident cells including macrophages and APC [127,128,129]. This may suggest that CD4+ T_RM_ sense their environment and induce the innate responses to fight off pathogens.

**Figure 2:**
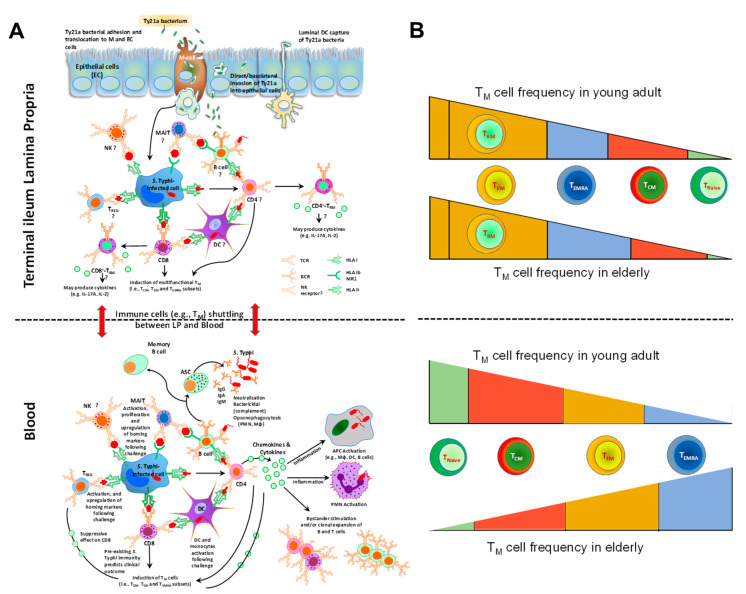
**Overview of the Immunity to *S*. Typhi in Peripheral Blood and the Gut as well as Age-related Changes in T-cell Subsets.** (**A**) Immunity to *S*. Typhi is extremely complex and the diagram summarizes our current knowledge and differences between mucosal and systemic responses to this microorganism. Multiple antigen-presenting cells (e.g., macrophages, dendritic cells, B cells) and effector cells (e.g., various effector and regulatory T-cell subsets, B cells, NK, and MAIT cells) are involved in the immune response. Red arrow indicates migration of specific cells between systemic and mucosal compartments. APC: antigen-presenting cells. ASC: antibody secreting cells. DC: dendritic cells. CD8: CD8^+^ T cells. CD4, CD4^+^ T cells. MAIT: mucosal associated invariant T-cells. Mφ: macrophages. NK: natural killer cells. PMN: polymorphonuclear neutrophil. T_M_: memory T-cells. T_CM_: central memory T cells. T_EM_: effector memory T cells. T_EMRA_: effector memory expressing CD45RA. T_reg_: regulatory T cells. HLA: human leukocytes antigen. HLA-I: HLA class I. HLA-II: HLA class II. BCR: B-cell receptor. TCR: T-cell receptor. MR1: HLA-I non-classical (b) molecule MR1. Ig: immunoglobulin. (**B**) Aging impacts T_M_ cell frequencies differently in tissues and blood. In the terminal ileum lamina propria, the frequencies of T_EM_, consisting mostly of T_RM_, do not seem to change with age. However, the frequencies of the other T_M_ subsets (T_EMRA_, T_CM_ and T_Naive_) appear to be impacted but only moderately. In blood, on the other hand, the differences in frequencies of the various T_M_ subsets are substantial. T effector memory (T_EM_, CD62L- CD45RA-); terminal effector memory T cells (T_EMRA_, CD62L- CD45RA+); T central memory (T_CM_, CD62L+ CD45RA-); naïve T cells (T_Naive_, CD62L+ CD45RA+). Figure adapted from [131].

It is also unclear which precursor gives rise to T_RM_ and whether CD4+ T_RM_ respond differently to pathogens than the peripheral CD4+ T effector subset. Our group recently reported that terminal ileum lamina propria mononuclear cells (LPMC) CD4+ T_RM_ subsets, obtained from Ty21a-vaccinated volunteers, contribute significantly to *S*. Typhi-specific IFN-γ, IL-17A, and IL-2 responses. Moreover, these responses differed in magnitude and characteristics between CD103+ and CD103- CD4+ T_RM_ subsets, suggesting a dichotomy in their contributions and possibly different roles in *S*. Typhi immunity (Figure 2A) [130]. Further, we showed that intraepithelial lymphocytes (IEL) CD4+ T_RM_ contributed significantly to *S*. Typhi-specific responses in the epithelium compartment. In contrast, in peripheral blood after Ty21a immunization, *S*. Typhi-specific CD4+ T-cell responses were mediated mostly by T_EM_ and CD45RA+T_EM_ (T_EMRA_) and, to a lesser extent, by T_CM_ [65,67]. CD4+ T_EM_ and T_EMRA_ subsets responses exhibited significant increases in *S.* Typhi-specific multifunctional (MF) cells post-vaccination mainly those IFN-γ and/or TNF-α. Meanwhile, IL-2, MIP-1β, IL-17A, and CD107a (cytotoxic surrogate marker) were produced in a small proportion of MF cells [67]. Altogether, these data suggest that immune responses to Ty21a vaccination are compartmentalized and that T_RM_ cells may be induced differentially at the site of infection than T_M_ in blood.

#### 3.3.2. CD8+ T_RM_

CD8+ T_RM_ constitute an important line of defense against pathogens mainly in mucosal tissues such as the intestine. CD8+ T_RM_ have a distinct genetic signature as defined by their cell surface expression of CD69, CD103, CD49a, and CD44. The vast majority of CD8+ T_M_ in peripheral blood are CD69-/CD103- whereas in the human intestine CD8+ T_RM_ are phenotypically characterized by their high expression of CD69 [132] and CD103 [113,114,115,116]. The functional hallmark of T_RM_ is its ability to remain in tissues and perform surveillance and in fact, CD8+ T_RM_ have been shown to persist in tissues for a long period of time [133,134,135]. Both CD103 and CD49a (integrin α1) are regulated on CD8+ T_RM_ to allow retention, mobility, and contribute to their development. CD49a form a heterodimer with CD29 (integrin β1) namely VLA-1 which is a collagen-binding integrin. Studies have shown that VLA-1 is not only critical for adherence to collagen IV (ColIV) but also for migration of cells along the collagen [136,137,138]. Finally, CD44, a C-lectin-containing glycoprotein is also a T_RM_ marker especially on CD8+T cells as it denotes previous activation and usually expressed on resting memory cells and newly generated effector cells [139]. Altogether, these markers indicate that the CD8+ T_RM_ population home to areas of epithelial surfaces (both the lamina propria and epithelium) which are common sites of infection. For example, CD8+ T_RM_ prefers to localize to ColIV-rich region because of high expression of CD49a whereas CD4+ T cells tend to flock to areas abundant in collagen 1 (Col1) [140]. Thus, understanding the function of surface receptors on T_RM_ may help to decipher their roles and location within tissues. Furthermore, the role of cytokines and chemokine in positioning T_RM_ within peripheral tissues is not well understood. Thus, different factors such as cytokines, chemokines, local antigens, and tissue-specific metabolites may be needed for the maintenance and differentiation of site-specific T_RM_, through complex signaling pathways and loops, which need to be unraveled.

Recent works in humans have tried to identify the transcriptional regulators of T_RM_, which would be critical for T_RM_ development and maintenance. T_RM_ subsets did not express the transcription factor (TF) Eomes and T-bet but seemed to express HOBIT [116]. However, HOBIT is found in both circulating and resident CD8+ T-cell populations in humans [113]. Hombrink et al., 2016 also identified NOTCH-1 and HIF1a in CD69+ CD8+ T_RM_ obtained from various tissues [116]. Organ specific transcriptional regulators have been identified in the T_RM_ subsets; however, most of these transcription factors do not appear to be “master regulators” of T_RM_ differentiation. Thus, T_RM_ differentiation and maintenance seems to be controlled by complex combinations of transcription factors.

CD8+ T_RM_ have been shown to mediate protective responses and maintain long-term immunity in various tissues and to diverse pathogens. For example, in human skin, CD8+T_RM_ induce specific immune responses to herpes simplex virus (HSV) [141,142]. Moreover, influenza-specific CD8+ T cells are maintained within the human lung T_RM_ subset [143,144], hepatitis B virus (HBV)-specific CD8 T cells are found within liver CD69^+^ memory T cells [145], and EBV specific CD8^+^ T_RM_ cells can be identified in spleen and tonsils [146]. The ability of T_RM_ to rapidly mount strong protective immunity to site-specific infection has led to the assessment of T-cell-mediated responses as part of vaccine development efforts. Our group has studied the effect of oral immunization with Ty21a on human terminal ileum LPMC and IEL CD8+ T_RM_. After immunization, terminal ileum LPMC and IEL *S*. Typhi-specific CD8+ T_RM_ and CD8+ CD103- T cells produced cytokines (e.g., IFN-γ, IL-17A and IL-2) either alone or in combinations (multifunctional) [147]. Taken together, these findings suggest site-specific generation, retention, and responses of CD8+ T_RM_ to pathogens.

CD8+ T_RM_ also seem to play a role in immunoregulation. Recent studies have shown that human CD8+ T_RM_ not only produce higher levels of cytokines (IFN-γ, IL-17A, TNF-α, and IL-2) associated with protective immunity but also higher levels of IL-10 than circulating T_EM_ cells [113,115,116,145]. Furthermore, CD8+ T_RM_ can potentially inhibit T-cell function as they express inhibitory proteins such as PD-1, LAG3, CTLA-4, and CD101 (inhibit proliferation) and exhibit decreased levels of Ki67 than circulating T_EM_ [113,146,148]. These observations suggest that CD8+T_RM_ can play roles in both protection against pathogens and in immune homeostasis, particularly at site of high antigenic content such as the intestine.

There is evidence for heterogeneity within human T_RM_ cells. For example, CD103 expression can vary on human CD8+ T_RM_ cells and CD103+ and CD103− T_RM_ cells are developmentally distinct subsets [113,149]. A recent study examining T_RM_ cells from human skin found that the CD49a+ CD103+ subset of CD8+ T_RM_ cells represented a subset with superior cytotoxic abilities compared to the CD49a- counterparts [150]. Our group recently reported that terminal ileum LPMC CD8+ CD69+ CD103+ T_RM_ exhibited significantly higher levels of *S*. Typhi-specific IL-17A in Ty21a-vaccinated than in unvaccinated volunteers (Figure 2A). In contrast, LPMC CD8+ CD69+ CD103- T cells showed significantly increased *S*. Typhi-specific levels of IFN-γ, IL-2, and IL-17A [147]. Our results suggest a dichotomy in *S*. Typhi-specific responses following Ty21a immunization. Thus, CD8+ T_RM_ show tremendous heterogeneity and may adopt tissue-specific functional or migration capacities, which will be an important area for future studies.

#### 3.3.3. Regulatory T Cells

Foxp3^+^ CD4^+^ regulatory T cells (T_regs_) are immune cells that contribute critically to the control of the immune system. Basically, T_regs_ are important regulators of immune responses including anti-pathogenic (inflammation) and anti-self (autoimmunity) and allergy. Circulating and lymphoid T_regs_ diversity, development, and function have been extensively studied in various animal models and humans and have been reviewed thoroughly elsewhere [151,152,153]. Recently, the role and importance of resident T_regs_ within non-lymphoid tissues have been recognized. These resident T_regs_ are critical for regulation of the kinetics and strength of immune responses but also regulation of non-immune cells [154,155]. For example, intestinal T_regs_ are different from other tissue T_regs_ as shown by colonic T_regs_ that preferentially expressed *Rorc*, a key T_H_17 lineage regulator encoding retinoic acid receptor-related orphan receptor *γ*t (RoR*γ*t) which is not present in other lymphoid tissue or splenic T_regs_ [156]. Therefore, tissue T_regs_ are adapted to their microenvironment and have functional specialization operating within the different tissues.

## 4. Respiratory Tract

### 4.1. Lymphoid Tissues in the Respiratory Track: Organization and Structures

In the upper respiratory tract (URT) of humans, most mucosal-associated lymphoid tissues are found in the Waldeyer’s ring that consists of the adenoid, tubal, palatine, and lingual tonsils [157]. In the Waldeyer’s ring, the structure and tight junctions utilized by the overlying lympho-epithelium are uniquely specialized in each site. For example, pseudostratified ciliated columnar epithelium is found in the adenoid, while stratified squamous epithelium predominates in tonsillar tissue [158]. Importantly, epithelial cells with some characteristics of M cells have been reported in human tonsils and adenoid [158,159]; however, evidence for their role as antigen transporting cells in the nasopharynx has been described only in mice [160]. Palatine tonsils, and possibly the adenoid, are highly efficient sites of antigen uptake and inductive sites for airway specific humoral immune responses as shown by a study that compared intratonsillar and intranasal vaccination (patients scheduled to undergo a tonsillectomy) with peroral and parenteral vaccination with tetanus and cholera toxin. The intratonsillar and intranasal routes were both efficient inducers of systemic specific antibodies, but mucosal immunity was conferred only to the upper airway, possibly due to preferential use of adhesion molecules and chemokines among the upper airway mucosal B cells.

In the URT, there is also an array of lymph nodes that are involved in sensitization and elicitation of airways responses including cervical, sublingual, and parotid nodes, as well as tracheobronchial nodes. Other lymphoid aggregates are also found in the human larynx epithelium (laryngeal associate lymphoid tissue-LALT-) and these persist into adulthood [161]. Bronchus-associated lymphoid tissues (BALT) are rarely present in healthy humans; however, they can be induced (iBALT) in chronic inflammatory airway disease or infections [162,163]. Given that lymphoid tissues in the respiratory track, particularly lymphoid aggregates (tertiary lymphoid aggregates) are not as organized as in the GALT (e.g., PPs), the question of whether these can support B-cell activation and differentiation has emerged. There is significant data showing several processes that take place in GCs are also present in the lymphoid aggregates. For example, various studies in allergic inflammation have shown that CSR can occur in the airways [164,165]. Studies have implicated DC-derived iNOS as an important enzyme driving CSR to IgA [166]. This is because, iNOS regulates expression of the TGFβ receptor, as well as the production of APRIL and BAFF, all of which play important roles in IgA class switching [166]. Considering that very large quantities of NO are produced in the respiratory system, especially in the sinuses and in the vicinity of NALT, this might be particularly important in the nasal cavity.

#### 4.1.1. Secretory IgA and Respiratory B Cells

One of the primary functions of B cells in the airways is the production of immunoglobulins, both within the parenchymal tissue and for exporting to the lumen of the airway. Airway B cells, like those in the gut, produce polymeric forms of IgA and IgM that are structurally maintained by the J-chain. Secretory IgA (sIgA) and sIgM contribute to “immune exclusion” in the respiratory track, which is similar to the one discussed in the gut. In humans, sIgA is the major immunoglobulin of the healthy upper and lower respiratory tract. Interestingly, IgD and IgG are also present and account for ~25% of nasal immunoglobulins [100]. The IgA1 subclass predominates in the upper and lower respiratory tract; however, nasal airway secretions typically have less IgA2 than bronchial secretions [167]. In humans, IgD comprises ~10% of nasally produced immunoglobulins. IgD is usually recognized largely for its presence on naïve B cells; however, a secreted form of IgD has been found in the human nasal mucosa and tonsils [168]. Secreted IgD is mainly produced by IgD+ IgM- B cells that have a PB phenotype. Interestingly, IgM-to-IgD class switching occurs via a non-canonical mechanism and can occur both in a T-dependent or T-independent mechanism, both of which require AID. Secreted IgD antibodies are usually polyreactive and recognize respiratory bacteria such as *Moraxella catarrhalis* and *Haemophilus influenza* [169].

In the airway, B cells activated in the NALT become PCs that preferentially switch to IgA and home back to the airway and to other mucosal sites [170]. Importantly, there is not a specific airway homing molecule like α4β7 integrin in the gut that has not been definitively identified. It has been suggested that homing to the airways may be dependent on α4β1 integrin and CCR10, whose ligands, VCAM-1 and CCL28, are expressed in the respiratory tract [170]. More recent studies have implicated CXCR3 as a marker to identify cells homing to the respiratory track [171].

#### 4.1.2. Tissue Resident Memory B Cells in Lungs

Onodera described in the lungs of influenza-infected mice a virus-binding B-cell population that expressed surface markers for splenic mature memory B cells (CD73, CD80, and CD273) along with CD69 and CXCR3 that are up-regulated on lung effector/memory T cells [171]. This lung B-cell population with memory phenotype persisted for more than 5 months after infection, and on reinfection promptly differentiated into PCs that produced virus-neutralizing antibodies locally. The production of local IgG and IgA neutralizing antibody was correlated with reduced virus spread [171]. Therefore, this population appeared to represent a type of tissue (lung) resident memory B cell. Later studies using parabiosis showed that lung-resident memory B cells (B_RM_ cells) were phenotypically and functionally distinct from their systemic counterparts. This study also showed that B_RM_ cells were established in the lung early after infection, in part because their placement required local antigen encounter. Moreover, lung B_RM_ cells, but not systemic memory B cells, contributed to early PBs responses following challenge (infection). Additionally, following secondary infection, antigen-specific B_RM_ cells differentiated in situ, whereas antigen-non-specific B_RM_ cells were maintained as memory cells. The data suggest that B_RM_ cells are an important component of immunity to respiratory viruses and suggest that vaccines designed to elicit B_RM_ cells must deliver antigen to the lungs [172]. More recent studies have suggested that lung-B_RM_ are induced also after pneumococcal exposure independent of the formation of tertiary lymphoid organs; however, these results need to be confirmed [173]. Whether lung B_RM_ cells are present in humans remains to be explored.

#### 4.1.3. B Cell Responses after Mucosal Immunization

The study of how different vaccine compositions affect the generation of B_M_ cells and LLPCs needs further study and influenza virus vaccination has allowed to start answering some questions. There are two kinds of seasonal influenza virus vaccines (1) live-attenuated influenza virus (LAIV) vaccines, and (2) inactivated influenza virus (IIV) vaccines. LAIV vaccines are delivered in the upper respiratory track (nasal mucosa); hence, these likely induce immune responses similar to those generated by natural infection. LAIVs have been used primarily in children. In this population LAIV vaccines boosted HA-specific IgG responses against the head and the full-length HA. Interestingly, in adults LAIV vaccines did not induce strong systemic antibody responses [174]. Two other studies with different avian pandemic LAIV vaccines (H5N1, H7N9) showed similar results. Interestingly, these vaccines were capable of inducing long-term immune memory, but this was revealed only after the administration of a matched IIV vaccine [175,176,177]. Studies in non-human primates (NHP) have tried unravelling how LAIV vaccines can prime these memory responses. These studies analyzed the B-cell responses systemically as well as in local lymphoid tissues [178] and found that the LAIV vaccine induced robust GCs in the mediastinal (lung-draining) lymph node. Moreover, after immunization, both HA-reactive PBs and B_M_ cells were found in the mediastinal lymph nodes and following intra-muscular (i.m.) immunization, these subsets were mobilized systemically. Therefore, it appears that LAIV vaccines prime local (mucosal) immune responses that are recalled systemically upon i.m. immunization.

### 4.2. Lung Tissue Resident Memory T (T_RM_) Cells

The lung represents a major site of antigenic entry site for viral and bacterial pathogens. Lung CD4 T_RM_ have been extensively characterized in mice models and shown to exhibit protective function. For example, the induction of CD4+ T_RM_ cells is influenced by the route of immunization as demonstrated by the two licensed influenza virus vaccines given by intranasal or parenteral routes in mice studies. FluMist (LAIV) administered intranasally generated CD4+ T_RM_ in the lungs resulting in long-term protection against other strains of influenza virus. However, Fluzone (IIV) did not induce CD4+ T_RM_ even when delivered parenterally or intranasally [179]. Similarly, immunization with SARS-CoV nucleocapsid (N) protein showed that intranasal but not subcutaneous administration of the vaccine elicits airway and lung-parenchymal antigen-specific memory CD4 T_RM_ cells in mice [180]. Interestingly, depletion of airway T_RM_ but not parenchymal resulted in loss of protection. In humans, these studies are not feasible; however, a recent study assessed the role of human lung memory T cells to *M. tuberculosis* (Mtb) infection using bronchoscopic antigen challenge with purified protein derivative of Mtb (PPD) in healthy volunteers with a positive PPD skin test. There was a significant increase in non-proliferative antigen-specific cells suggesting that lung CD4+ T_RM_ might have migrated to the airways in response to antigen challenge [181]. These results altogether show the importance of lung CD4+ T_RM_ in respiratory infections, and the importance of harvesting their potential during immunization.

In addition, following influenza infection, it was shown that late antigen recognition by CD4+ T cells resulted in autocrine IL-2 production which sustained the maintenance of CD4+ T_RM_ cells in the lung [182] with a transcriptional signature distinct from that of circulating CD4+ T-cell populations but similar to that of CD8+ T_RM_ [125,183]. Maintenance of both naïve and lymphoid homing CD4+ T_M_ cells requires IL-7 signaling [126]. Interestingly, higher levels of IL-7R were expressed by lung T_RM_ than circulating effectors following influenza infection. Circulating CD4+ T cells that are treated with Fc-fused IL-7 can be recruited into the lungs and acquire T_RM_ like phenotype and contribute to secondary immune responses [184].

Likewise, CD8+ T cells (potentially T_RM_) specific for influenza and respiratory syncytial virus are found in higher frequencies within human lungs than in the spleen, blood, and skin [144,185]. The role of CD8+ T_RM_ in the lung is still unclear and importantly, the mechanisms by which both CD4+ and CD8+ T_RM_ protect the lung have not yet been deciphered. Initial studies have shown the importance of production of cytokines, such as IFN-γ, in the lung to mediate protection against influenza virus [186,187]. However, IFN-γ production was not associated with the protection to Mtb [188]. Altogether, these studies point out that lung T_RM_ cells are important in generating protective immunity against respiratory pathogens and suggest that it would be beneficial to generate and maintain T_RM_ in the lung to provide optimal protection.

## 5. Age-Associated Changes in the B- and T-Cell Compartments

**Figure 3:**
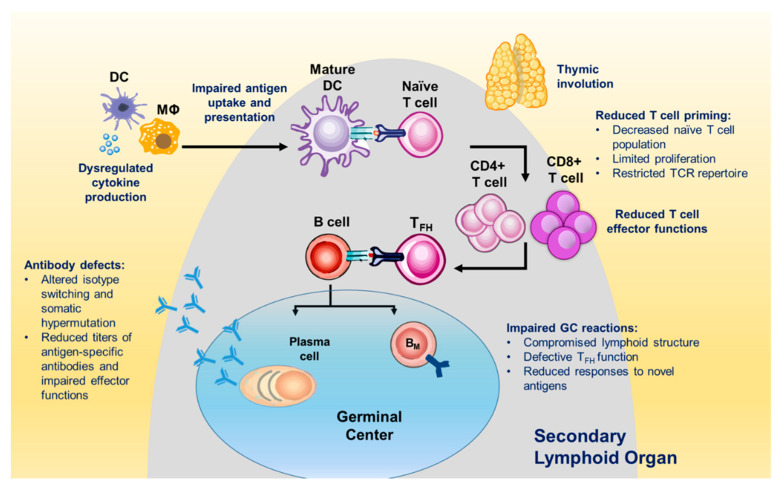
**Overview of Age-Associated Impairments in the Immune System.** Multiple compartments are affected by immunosenescence, including the innate and adaptive responses. Figure adapted from [196].

Currently, there are 700 million individuals over the age of 65 years worldwide. Population projections suggest that this population will double in size by year 2050 [189]. A series of age-associated changes to the immune system, referred to as immunosenescence, contribute to the increased vulnerability to infections among older adults (>65 years) (Figure 3). Immunosenescence results in impaired humoral and cellular responses to pathogens [190] and as a consequence, compared to younger individuals, older adults often experience long-term sequelae and increased morbidity [191]. For example, influenza, respiratory syncytial virus (RSV), herpes zoster (HZ) or shingles, pneumococcal disease, and foodborne illnesses disproportionately affect the elderly [192,193,194,195].

### 5.1. Impact of Aging on B Cells

B cells are affected by aging. For example, aged individuals vaccinated with tetanus, diphtheria, and two pertussis antigens elicited lower antibody levels than younger adults [197,198]. Additionally, a booster dose at age 60 still failed to elicit protective levels of antigen-specific antibody levels, further highlighting the decline in humoral immunity with age [197]. Many studies involving influenza virus vaccines have shown that older adults overwhelmingly exhibit lower seroconversion rates and protective HAI titers [199]. Specific mechanisms responsible for B-cell intrinsic defects remain to be completely understood; however, several phenotypic differences among B-cell subsets in aged individuals have been reported. For example, an accumulation of CD27+ B cells has been described, as well as a decrease in PC differentiation [200]. There is an increase in frequency of class-switched B_M_ cells with age in comparison to B cells that have yet to encounter antigen. Interestingly, in comparison to younger adults immunized with consecutive influenza vaccines, older individuals showed similar frequencies of B_M_ cells and PBs. However, HA-specific serum titers and BLIMP-1 expression levels were severely reduced in the aged group, which suggested a defect in the ability of B_M_ cells to differentiate into PCs [201]. In addition, individuals aged 86+ years have much lower BCR diversity, in comparison to younger adults [202]. The reduced pool of naïve B cells available in older individuals likely restricts the number of possible clones able to respond to novel antigens. Indeed, aged individuals receiving influenza virus vaccine show hypermutated IgG but fewer responding B-cell clones in comparison to adults [198]. Importantly, vaccine-induced antibodies failed to elicit optimal effector functions that are normally induced in younger adults. Specifically, reduced protective HAI titers following trivalent influenza vaccination and lower opsonization activity in the case of the 23-valent pneumococcal polysaccharide vaccine (PPV23) are observed in older individuals [199,203]. Therefore, even when sufficient antibody titers are generated, the protective capacity of the vaccine-induced antibodies may be significantly reduced in the aged population.

Studies in aged mice have demonstrate AID-associated impaired CSR and decreased effector antibody isotypes [204]. Furthermore, a decline in AID mRNA in human B cells correlates with decreased antibody affinity maturation following influenza vaccination has been reported [205]. Interestingly, a subset of mature B cells was identified in aged mice. These cells, termed age-associated B cells, were responsive to innate stimuli but resistant to BCR and CD40 stimulation [206,207]. B cells with a similar profile have been identified during autoimmune diseases and viral infections in humans; [206,208] however, these cells were part of the DN compartment, showed characteristics of exhausted B cells and were present at similar rates in young adults and older individuals [209]. Therefore, as of today, age-associated B cells remain a controversial population in humans.

### 5.2. Impact of Aging on T Cells

The T-cell compartment is affected by the aging process by the contraction of the naïve T (CD4+ and CD8+) cell repertoire [210] and expansion of terminally differentiated cell subsets with altered effector functions [211]. However, human studies have shown that age impact on the diversity of naïve T cell repertoire is not linear. For example, until age 70, CD4+ T-cell diversity is maintained relatively stable at ~2 × 10^7^ different T-cell receptor β-chains but after age 70, CD4+T diversity decreases significantly to 2 × 10^5^ specificities [212]. In addition, the capacity of T cells to proliferate is significantly reduced by age 70 [213]. Our current understanding of human immunosenescence is primarily derived from the study of peripheral blood cells. Studies focusing on age-associated changes in the tissue microenvironment are limited. Interestingly, there are substantial differences in T-cell frequencies, function, and activation status between tissues (e.g., lungs, intestine) and peripheral blood.

In the lungs, the composition of T cells also evolves with age. During early age, the predominant population in the lungs are naïve T cells and T_regs_, which play an important role in promoting tolerance during infancy [214,215]. During late childhood, there is a shift in cell populations since the T_EM_ cell subset becomes the major population in the lungs and these cells subsequently acquire a T_RM_ phenotype [114,215,216]. Throughout many decades of adulthood, the predominance of T_RM_ among lung T cells is stable [216]. However, it is unclear how T_RM_ organized within the complex structures of the lung and how T_RM_ functional capacity is impacted with age.

In the human intestine (terminal ileum), the majority of CD8+ T cells are part of the T_RM_ subset, while blood CD8+ T cells are composed of a significant fraction of naïve and T_CM_ subsets [147]. So, how does the aging process influence tissue CD4+ and CD8+ T cells? A recent study showed that the dynamics of blood CD8+ T-cell activation and homeostatic proliferation were not reflected in matched rectosigmoid colonic CD8+ T cells [217]. Moreover, age-associated differences in naïve and memory CD4+ T-cell frequencies in human spleen and lymphoid tissues were not observed in multiple intestinal tissues sites [216]. Using an aging mouse model, it has been demonstrated that CD4+ T cells concurrently accumulate in the small intestine and colonic lamina propria and PPs while being depleted in the spleen and LN [218]. Our group examined and compared the responses elicited by Ty21a-immunization on terminal ileum LPMC T_M_ and T_RM_ subsets isolated from biopsies of elderly and adult volunteers. We showed that aging influences several immune parameters, including (i) the frequencies of CD4+ T_RM_ subsets (Figure 2B); (ii) the CD4/CD8 ratio, which was found to be different in terminal ileum LPMC than in blood; (iii) the levels of cytokine production following Ty21a immunization by *S*. Typhi-specific LPMC CD4+ and CD8+ T_RM_ cells (Manuscript under review). Altogether, these data indicate that the process of immunosenescence may differ between peripheral blood and tissues in terms of cell frequencies and phenotypic characteristics. These results highlight the importance of assessing immunity at the local level (e.g., lung, intestine) and suggest that further studies in humans are needed to evaluate the immune response context of infectious diseases and aging.

## 6. Conclusions

Most studies of the human immune responses to infection/vaccination have used blood specimens; however, recent studies have started to explore the immune response in diverse tissues and have revealed a profound anatomic compartmentalization of B- and T-cell subsets. Local immune responses, particularly in mucosal surfaces that are exposed to high concentrations of foreign antigens (e.g., intestines and respiratory track), play multiple roles including defense against invading microorganisms and homeostasis maintenance of the local environment. Initial studies have advanced our understanding of the factors involved in priming, homing, and long-term maintenance of these cells at local sites; however, we still do not have a complete picture of these processes. Future studies focusing on single cell level detailing the molecular, phenotypic, and functional profiling of tissue immunity will provide tremendous insights in the immunopathological processes. In addition, studies to explore the mechanisms involved in tissue specialization of B- and T-cell responses such as epigenetics and miRNA will help us decipher how the immune system operates in tissues during infection and homeostasis. This information can be critical for the development of novel vaccines and therapeutics that target local immune cells. Finally, it is known that the immune system undergoes several changes during the life-span of an individual. However, age-related changes in tissues appear not to be reflected in peripheral blood, suggesting that more detailed studies of the immune system at the local level, across multiple age groups, are needed. As the global population ages, a better understanding of age-related alterations of the immune response are becoming critical to limit infections and design vaccines tailored for diverse age groups.

## Data Availability

Not applicable.
